# Incidence and Predictors of Drug-Induced Liver Injury in Pediatric Tuberculosis Patients Under Anti-tubercular Therapy: A Prospective Observational Study

**DOI:** 10.7759/cureus.85661

**Published:** 2025-06-09

**Authors:** Vasanthakumar Manokaran, Baljinder Kaur, Jane Allen Christa A, Reshma I

**Affiliations:** 1 Department of Pediatrics, Vinayaka Mission's Medical College and Hospital, Vinayaka Mission's Research Foundation Deemed to be University (DU), Karaikal, IND; 2 Department of Pediatrics, Government Medical College, Baba Farid University of Health Sciences, Patiala, IND

**Keywords:** anti-tubercular therapy, drug-induced liver injury, liver function monitoring, malnutrition, pediatrics, tuberculosis

## Abstract

Background: Tuberculosis (TB) remains a significant global health challenge, particularly in pediatric populations, where effective treatment with anti-tubercular therapy (ATT) is often complicated by adverse drug reactions. Drug-induced liver injury (DILI) is among the most serious complications of ATT, and identifying risk factors for DILI in children is essential for improving treatment safety and outcomes.

Objective: This study aimed to determine the incidence of DILI in pediatric TB patients undergoing ATT and identify demographic and clinical factors associated with its development.

Methods: A prospective observational study was conducted over 18 months at a tertiary care center in South India. Fifty children aged 1-14 years diagnosed with TB and initiated on ATT were enrolled. Liver function tests (LFTs) were performed at baseline, one month, and six months, and clinical parameters were monitored to identify DILI cases. Nutritional status was assessed using WHO growth standards, and statistical analyses were conducted to identify significant risk factors.

Results: DILI was observed in 16 of 50 patients (32%). Malnutrition was present in 70% of DILI cases compared to 48% of non-DILI cases (p < 0.05). Female patients showed a higher incidence of DILI (56%) than males (44%). Baseline liver enzyme levels, specifically serum glutamic-oxaloacetic transaminase (SGOT) and serum glutamic-pyruvic transaminase (SGPT), were significantly higher in patients who developed DILI (p < 0.05). The most common clinical presentation of DILI was jaundice (50%), followed by anorexia and abdominal pain. Pulmonary TB accounted for 50% of DILI cases, while CNS TB represented 37.5%.

Conclusions: DILI is a common complication of ATT in pediatric TB patients, with malnutrition, female gender, and elevated baseline liver enzymes identified as significant risk factors. Routine liver function monitoring and nutritional interventions should be integral to TB management in children to mitigate the risk of DILI and improve treatment outcomes.

## Introduction

Globally, India is responsible for 28% of the worldwide tuberculosis (TB) load among the six high-burden nations in Southeast Asia, with pediatric cases forming a substantial portion of the population affected by this disease [1,2]. Additionally, its burden is the greatest in the world. The National TB Prevalence Survey 2021 found that 31.3% of people over 15 had a crude prevalence of TB infection and that 5-10% of those who have TB infection subsequently develop clinically active TB [3,4]. Drug-induced liver injury (DILI) is one of the most concerning adverse effects of anti-tubercular therapy (ATT), leading to treatment interruptions and potentially severe outcomes. The incidence of DILI among TB patients varies widely, ranging from 2% to 28%, depending on study settings and populations [5]. However, in children, the pathophysiology of DILI is compounded by immaturity of hepatic enzyme systems, differences in drug metabolism, and frequent co-existing malnutrition, especially in resource-limited settings like India [6].

Although DILI has been extensively studied in adult TB patients, there is a paucity of data focusing on pediatric populations. Children are particularly vulnerable due to distinct pharmacokinetic and pharmacodynamic profiles, which influence drug metabolism and toxicity. In addition, malnutrition, a common issue in pediatric TB patients, further exacerbates the risk of hepatic injury. Identifying factors contributing to DILI in children is critical for improving the safety and efficacy of ATT [7,8].

This study investigates the incidence and predictors of DILI in pediatric TB patients undergoing ATT. A prospective observational study was conducted on 50 children aged 1-14 years diagnosed with TB, where clinical and biochemical parameters were monitored at baseline, one month, and six months. The study aimed to identify demographic and clinical predictors, such as malnutrition and TB type, associated with DILI in this vulnerable population. The results of this study provide insights into the burden of DILI in pediatric TB patients and highlight key predictors such as malnutrition and gender differences. These findings underscore the need for routine liver function monitoring and integrated nutritional support to mitigate the risks of DILI and improve treatment outcomes for children with TB.

## Materials and methods

This prospective observational study was conducted over a period of 18 months, from January 2022 to June 2023, at a tertiary care center in North India. The study population included children aged 1-14 years with a confirmed diagnosis of TB. Both pulmonary and extrapulmonary forms of TB were included. Patients were required to meet the inclusion criteria, which mandated that they be newly diagnosed with TB and initiated on ATT according to the Revised National Tuberculosis Control Program (RNTCP) guidelines. Written informed consent was obtained from the parents or guardians of all participants. Exclusion criteria included pre-existing liver diseases, concurrent use of hepatotoxic medications, and the presence of systemic illnesses that could influence liver function. Additionally, patients who were lost to follow-up or had incomplete clinical data were excluded from the study. Ultimately, a total of 50 pediatric TB patients were enrolled in the study.

All enrolled patients received standardized anti-TB treatment according to national guidelines. Table 1 presents the drug regimen. Dosages were precisely calculated based on individual body weight to optimize therapeutic efficacy while minimizing the risk of adverse effects.

**Table 1:** Standardized anti-TB treatment received by the patients

Drug	Dose (mg/kg/day)	Average Dose (mg/kg/day)	Maximum Dose (mg)
Rifampicin	10–20	15	600
Isoniazid	7–15	10	300
Pyrazinamide	30–40	35	2,000
Ethambutol	15–25	20	1,500
Streptomycin	15–20	20	1,000

Data collection involved a comprehensive baseline assessment, including detailed history-taking, clinical examination, and biochemical evaluation. Presenting symptoms, nutritional status, and co-morbidities were recorded during the initial visit. Anthropometric measurements, such as weight and height, were taken to assess malnutrition, which was classified according to the World Health Organization (WHO) growth standards. Liver function tests (LFTs), including serum glutamic-oxaloacetic transaminase (SGOT), serum glutamic-pyruvic transaminase (SGPT), alkaline phosphatase (ALP), and total serum bilirubin (TSB), were performed before the initiation of ATT to establish baseline values.

Follow-up evaluations were conducted one month and six months after the commencement of ATT. These assessments included repeated LFTs and clinical evaluations for signs and symptoms of hepatotoxicity, such as jaundice, anorexia, abdominal pain, or lethargy. Nutritional status was reassessed during follow-ups to monitor changes in weight and growth during treatment. DILI was defined according to the American Thoracic Society and WHO criteria, with serum SGOT or SGPT levels elevated more than five times the upper limit of normal (ULN) without symptoms or more than three times the ULN with symptoms. Elevation of TSB levels greater than two times the ULN in conjunction with jaundice was also considered diagnostic of DILI. Malnutrition was assessed using WHO standards for weight-for-age and height-for-age z-scores. Severe malnutrition was defined as a weight-for-age z-score below -3 standard deviations or the presence of clinical signs of wasting. This classification was essential for determining the relationship between nutritional deficits and the risk of developing DILI.

The study protocol received ethical clearance from the Institutional Ethics Committee, Government Medical College, Patiala, Punjab, India, with reference number BFHUS/2K21p-TH/1482.

For statistical analysis, descriptive statistics, including means, standard deviations, and percentages, were used to summarize demographic and clinical characteristics. Categorical variables, such as gender, age groups, and nutritional status, were compared between DILI and non-DILI groups using the chi-square test. Continuous variables, such as liver enzyme levels, were compared using independent t-tests. Data analysis was performed using IBM SPSS Statistics for Windows, Version 25 (Released 2017; IBM Corp., Armonk, New York), and a p-value of less than 0.05 was considered statistically significant.

## Results

Study population and baseline characteristics

A total of 50 pediatric patients diagnosed with TB and undergoing ATT were included in the study. The mean age of the participants was 10.5 ± 3.2 years, with the majority falling within the 11-14 years age group. The gender distribution showed that 28 participants (56%) were female, while 22 participants (44%) were male. Malnutrition was observed in 26 (52%) of the total cohort, highlighting the prevalence of nutritional deficiency. Regarding the type of TB, 23 (46%) patients had pulmonary TB, while 27 (54%) were diagnosed with extrapulmonary TB (Table 2).

**Table 2:** Baseline characteristics of the study population

Characteristic	Value
Total participants	50
Mean age (years)	10.5 ± 3.2
Male (%)	44
Female (%)	56
Malnutrition (%)	52
Pulmonary TB (%)	46

Incidence of DILI

DILI was observed in 16 (32%) of 50 patients. Among these cases, females were disproportionately affected, accounting for nine (56%) of the DILI group, compared to seven (44%) males. Age-wise, the highest incidence was observed in the 11-14 years group, constituting 10 (62.5%) of the DILI cases, followed by the 6-10 years group (n=5, 31.2%) and the 1-5 years group (n=1, 6.25%).

Biochemical parameters in DILI and non-DILI cases

Biochemical analysis revealed significant differences in liver enzyme levels between DILI and non-DILI groups. Patients with DILI exhibited markedly elevated levels of SGOT, SGPT, ALP, and TSB compared to those without DILI. The mean SGOT level in DILI cases was 223.62 ± 383.76 U/L, while non-DILI cases showed a mean level of 36.06 ± 23.36 U/L. Similarly, the mean SGPT level was 210.50 ± 366.28 U/L in DILI cases, compared to 29.94 ± 27.01 U/L in non-DILI cases. TSB levels were also significantly higher in the DILI group, with a mean value of 1.00 ± 0.92 mg/dL, compared to 0.53 ± 0.34 mg/dL in the non-DILI group (Table 3).

**Table 3:** Biochemical parameters in DILI and non-DILI cases DILI: drug-induced liver injury; SGOT: serum glutamic-oxaloacetic transaminase; SGPT: serum glutamic-pyruvic transaminase; ALP: alkaline phosphatase; TSB: total serum bilirubin; SD: standard deviation

Parameter	DILI Cases (Mean ± SD)	Non-DILI Cases (Mean ± SD)
SGOT (U/L)	223.62 ± 383.76	36.06 ± 23.36
SGPT (U/L)	210.50 ± 366.28	29.94 ± 27.01
ALP (U/L)	181.02 ± 131.70	163.74 ± 85.05
TSB (mg/dL)	1.00 ± 0.92	0.53 ± 0.34

**Figure 1:**
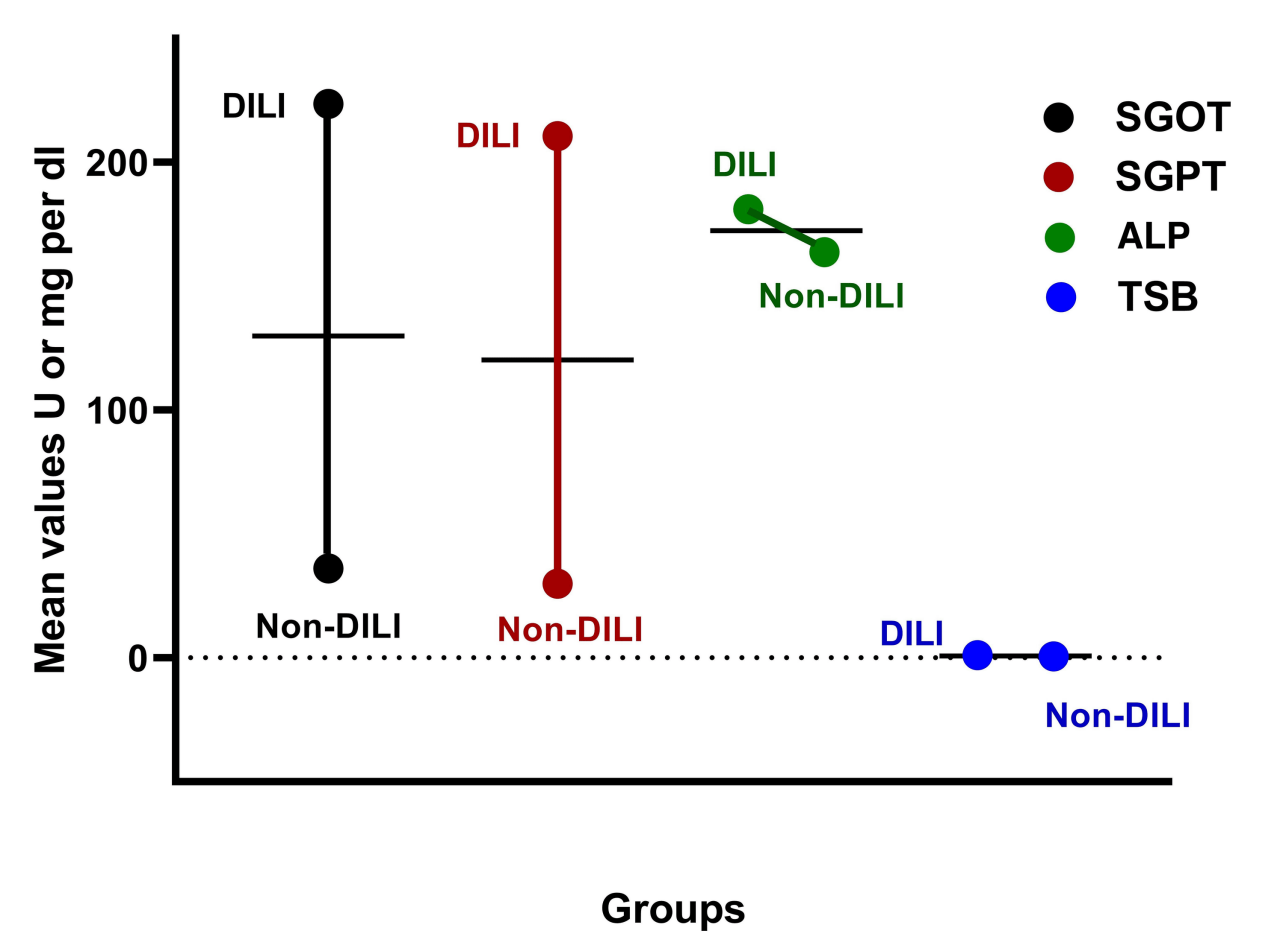
Trends in liver enzyme levels across DILI and non-DILI groups, highlighting the stark differences in SGOT, SGPT, ALP, and TSB. DILI: drug-induced liver injury; SGOT: serum glutamic-oxaloacetic transaminase; SGPT: serum glutamic-pyruvic transaminase; ALP: alkaline phosphatase; TSB: total serum bilirubin

Figure 1 displays the comparative biochemical parameters, including SGOT, SGPT, ALP, and TSB between DILI and non-DILI groups, highlighting significant elevations in DILI cases.

Risk factors associated with DILI

Malnutrition was a prominent risk factor associated with DILI. Among the DILI cases, 11 (70%) patients were malnourished, compared to 16 (48%) patients in the non-DILI group. Additionally, the type of TB appeared to influence the likelihood of developing DILI. Pulmonary TB accounted for eight (50%) of DILI cases, while six (37.5%) were associated with CNS TB.

**Figure 2:**
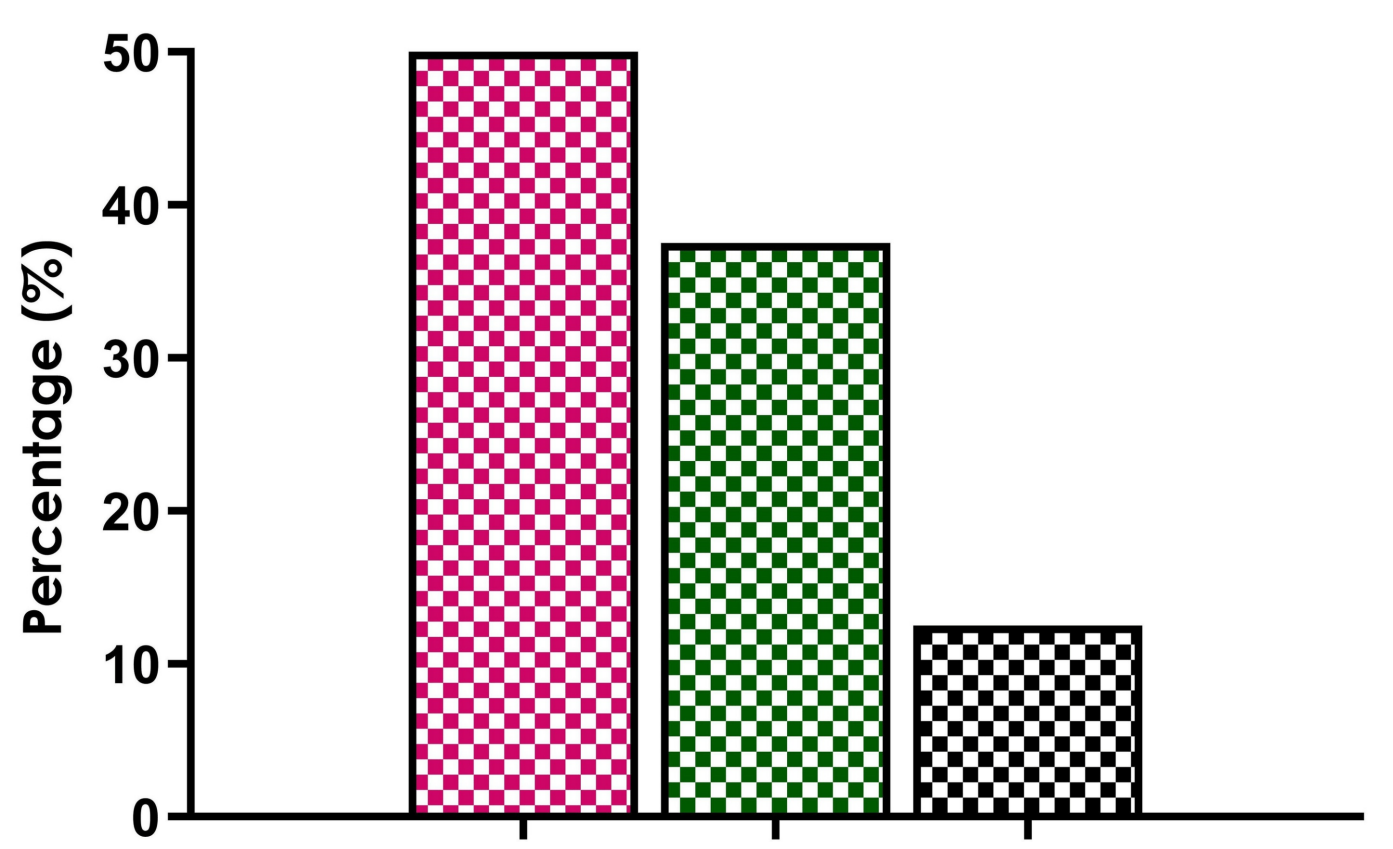
The distribution of DILI cases across TB types— the proportion of pulmonary TB, CNS TB, and other case types. DILI: drug-induced liver injury; SGOT: serum glutamic-oxaloacetic transaminase; SGPT: serum glutamic-pyruvic transaminase; ALP: alkaline phosphatase; TSB: total serum bilirubin

Figure 2 illustrates the distribution of DILI cases across different types of TB, with the pink bar representing pulmonary TB, the green bar representing CNS TB, and the black bar representing other TB types.

Clinical manifestations of DILI

The clinical manifestations of DILI included jaundice, anorexia, abdominal pain, and lethargy. Jaundice was the most common presentation, observed in eight (50%) of the DILI cases. Other symptoms were less frequent but contributed to the overall clinical burden of the condition.

## Discussion

The difficulty in treating TB is that it requires a lengthy course of treatment, repeated drug administration, and associated toxicities. Numerous medications and host-related factors have been linked to an elevated risk of anti-TB drug-induced hepatotoxicity over the course of decades of clinical observation. This study highlights a significant incidence of DILI among pediatric TB patients undergoing ATT. The observed incidence of 32% is within the range reported in previous studies on ATT-induced hepatotoxicity, which varies between 5% and 33%, depending on the population and setting [9]. The findings of the present study also corroborate earlier reports on ATT-induced DILI. A study from China reported an incidence rate of 27% in children treated with ATT, emphasizing the burden of hepatotoxicity in pediatric populations [10].

One of the principal roles of the liver is the biotransformation of various nutrients, medications, and supplements into compounds that can be securely absorbed, metabolized, and excreted by the human body. However, it cannot metabolize all the compounds safely or beneficially, resulting in DILI through a diversity of mechanisms. There are myriads of factors, including malnutrition, gender, and elevated baseline liver enzyme levels, that were identified as significant risk factors contributing to hepatotoxicity [11-14]. In the present study, malnutrition was a prominent risk factor associated with DILI, where 70% of patients were malnourished compared to 48% in the non-DILI group. These findings were in accordance with a systematic review that documented malnutrition as a key risk factor for DILI, highlighting that poor nutritional status exacerbates hepatic vulnerability due to deficiencies in essential nutrients required for drug metabolism [7]. Another study from India found that children with malnutrition were two times more likely to develop DILI compared to their well-nourished counterparts [15].

All tissues and organs can be affected by adverse medication responses, but liver damage is one of the most dangerous. Numerous investigations have verified that female individuals are generally at a higher risk of adverse drug reactions than males. The overwhelming body of evidence indicates that women seem to be more vulnerable than men to liver stress, particularly in reaction to different classes of some medicines, even if DILI is rare. This sex variation in vulnerability to drug-induced liver damage has been explained by a variety of theories. Collectively, these theories point to three fundamental sex-dependent processes, such as variations in different facets of drug pharmacokinetics or pharmacodynamics after specific medications are administered [16]. The gender distribution in this present study showed that 56% of the participants were female, while 44% were male. This aligns with earlier studies that reported a female predominance in DILI cases during ATT, potentially due to differences in hepatic enzyme activity and hormonal influences [17]. The elevated baseline liver enzyme levels in DILI cases observed in this study are also consistent with earlier findings that emphasized the predictive value of baseline LFTs in identifying high-risk patients [18]. Gender-specific monitoring strategies may also be warranted, given the higher susceptibility of females to DILI. Tailoring drug regimens based on individual risk profiles, particularly in patients with pre-existing hepatic vulnerabilities, could enhance treatment safety. Adjusting the doses of hepatotoxic drugs like isoniazid and pyrazinamide or opting for drug-sparing regimens in high-risk patients may reduce the incidence of DILI without compromising therapeutic efficacy [9].

When it comes to managing drug-induced liver damage, the primary treatment is to stop using the drugs or medications that are probably causing it. Most cases of drug-induced liver damage resolve on their own without the need for special supportive care or treatment. When determining the causality of DILI, this feature of spontaneous recovery after stopping offending drugs is a crucial requirement. Furthermore, DILI still presents as a difficult condition with unique diagnostic and therapeutic challenges. Despite this, numerous unanswered questions remain regarding the etiology of diseases and risk factors that require further research. Beyond the current determinants, there is also an obvious need for new biomarkers and outcome predictors. Another challenging scenario is presented by the introduction of new treatment modalities, especially oncological immune modulators, for which physicians should be aware of the possibility of hepatotoxicity [19]. Additionally, addressing malnutrition through the supplementation of essential nutrients and proteins can mitigate hepatic stress and improve drug metabolism, as supported by recent studies [20-22]. The high incidence of DILI underscores the importance of incorporating routine liver function monitoring into pediatric TB management protocols. Early detection through regular LFTs could prevent severe hepatotoxicity and reduce treatment interruptions. The findings also underscore the importance of incorporating nutritional interventions into TB treatment.

Strengths and limitations

This study provides valuable insights into the burden of DILI in pediatric TB patients in a high-burden setting. The prospective observational design enabled real-time data collection and monitoring, thereby minimizing recall bias. However, the single-center nature of the study and the relatively small sample size limit the generalizability of the findings. The lack of genetic analysis, such as screening for NAT2 and CYP450 polymorphisms, represents a missed opportunity to explore the role of pharmacogenomics in DILI susceptibility. Additionally, the absence of HIV co-infection data may have overlooked an important confounding factor, as co-infection is known to increase hepatotoxicity risk.

Future directions

To address these limitations, future studies shall adopt a multicenter approach with larger sample sizes to validate the findings and explore additional risk factors. Generally, DILI in children is rare, and clinical trials typically do not include enough patients to identify risk factors. To further understand the risk factors for children's DILI, multicenter collaborative initiatives at the international level should be done, as should more stringent post-marketing surveillance measures, such as requiring the reporting of DILI cases following initial marketing approval. Longitudinal studies with extended follow-up periods could capture late-onset DILI cases and provide a more comprehensive understanding of its temporal patterns. Incorporating pharmacogenomics testing into TB treatment protocols could pave the way for personalized medicine, enabling tailored drug regimens that minimize hepatotoxicity risks while maintaining efficacy.

## Conclusions

This study identifies a substantial burden of DILI in pediatric TB patients undergoing ATT, with malnutrition, female gender, and elevated baseline liver enzymes emerging as significant risk factors. The findings suggest the need for routine liver function monitoring, targeted nutritional interventions, and personalized treatment strategies to improve the safety and efficacy of TB management in children. Children may be more or less susceptible to DILI than adults, depending on the medicines involved. It is unclear, therefore, if there are any notable differences between children and adults in terms of DILI. Children's DILI is not well understood based on the available mechanistic data, which are mostly derived from studies in adults. Perhaps as a result of the dearth of data, there are no regulatory guidelines in place to address children with DILI. In order to properly utilize potentially hepatotoxic medications in children, further research is required to better forecast the likelihood of DILI in children and identify its risk factors.
